# The Microscope and the Tubular System: Trend or Real Benefit in Lumbar Spinal Stenosis and Disc Herniation?

**DOI:** 10.7759/cureus.99980

**Published:** 2025-12-23

**Authors:** Antonio Sosa Najera, Alejandro Ceja, Raul Huato Reyes, Tommy C Junior Quispe Pari, Carlos Morales Valencia

**Affiliations:** 1 Neurosurgery and Spine Surgery, Hospital Angeles Morelia, Morelia, MEX; 2 Neurosurgery and Spine Surgery, Hospital Star Médica Morelia, Morelia, MEX; 3 Neurosurgery and Spine Surgery, Guanajuato Biomedical Hospital, Guanajuato, MEX; 4 Vascular Neurosurgery, Instituto Nacional de Neurología y Neurocirugía, Mexico City, MEX; 5 Neurological and Spine Surgery, Universidad Autónoma del Estado de México, Toluca, MEX

**Keywords:** disc herniation, herniated nucleus pulposus, lumbar spinal stenosis, spinal canal stenosis, spine microsurgery, spine stenosis, spine surgery

## Abstract

The use of the microscope in minimally invasive spinal surgery has contributed to improvements, as it provides the surgeon with a better view of the surgical field, due to the magnification, depth, and better illumination of anatomical structures of vital importance for the patient's functionality, and also supports the ergonomics of the patient and surgeon within the surgical act. Tubular microdiscectomy is a minimally invasive procedure that works through a small incision, with less injury to the paraspinal muscles, less tissue retraction, and, therefore, less probability of producing postoperative infections. It can be applied in different pathologies of the lumbar spine and is an effective surgical technique for the treatment of lumbar disc pathology and spinal stenosis, with the benefits of minimally invasive spine surgery.

## Introduction

The techniques used for spine surgery have evolved over time. Since A.G. Smith reported the first laminectomy in 1829, spine surgery has evolved to include different surgical techniques. Open lumbar microdiscectomy was described by Caspar and Yasargil in 1977 [1,2].

In 1991, Faubert and Caspar performed and described an approach to lumbar spine surgery via a tube system [3]. The microscope was first used in 2003, offering greater benefits to patients. Microscopes have contributed to improvements in minimally invasive spine surgery because their magnification provides surgeons with a better visualization of the surgical field, as well as better depth and illumination of anatomical structures [4,5]. For some time, some authors have considered the use of microscopes to be a contributing factor to contamination. On the other hand, several studies have advocated the use of this tool due to its capacity to improve visualization for the entire surgical team [6,7].

The microscope is often replaced by loupes with frontal light in surgical practice. A retrospective study by Omar et al. [8] evaluated and compared the use of front-lighted surgical loupes with the use of microscopes for lumbar spine surgery, concluding that compared with front-lighted surgical loupes, microscopes do not increase surgical site infection rates but significantly increase the likelihood of longer operative times. Kumar et al. compared the use of loupes with frontal light and microscopes in lumbar spine surgeries and found the microscope to be superior in terms of reducing postoperative complications [9].

Tubular microdiscectomy is a minimally invasive procedure that involves a small incision, causing less injury to the paraspinal muscles, less retraction of the tissues, and, therefore, a lower probability of postoperative infections [10]. This procedure can be performed for different pathologies of the lumbar spine and is effective for treating lumbar disc pathology and spinal stenosis [11-13].

The objective of this work was to identify complications associated with the unilateral tubular approach to posterior lumbar decompression using a microscope in patients with a diagnosis of narrow lumbar canal and lumbar disc herniation; additionally, this work aimed to evaluate the quality of life of patients in the short and long term by measuring pain relief with the visual analog pain scale (VAS) and the Oswestry Disability Index [14,15]. We hypothesized that, because this approach involves a small incision through a tubular system, there would be less retraction of the paraspinal muscles when combined with the use of a microscope due to improved visualization of the anatomical structures. We also proposed that there would be a reduced risk of complications such as dural tears and improved quality of life as measured via the performance of normal daily activities and postoperative pain.

## Materials and methods

An observational, ambispective analytical study was performed with a sample of 54 consecutive patients who presented with a diagnosis of a narrow lumbar canal and disc herniation. Patients were sampled from a high-volume spine surgery hospital from January 2021 to January 2024 and were scheduled for surgery using a unilateral tubular system combined with an operating microscope. Data were obtained only from the patients’ clinical records and included sex, age, spinal levels that were operated on, VAS score, Oswestry Disability Index score, length of hospital stay in days, surgical time, number of procedures, and complications.

All patients between 18 and 90 years of age, patients with clinical and radiological diagnoses compatible with lumbar spinal stenosis and disc herniation, and patients with little or no response to conservative treatment for at least two months were included. Patients were excluded if they had undergone previous operations, or had not undergone surgery via the tubular technique or with a surgical microscope, were under 18 years of age, or had other spinal pathologies. All the procedures were performed by a spinal surgery specialist who is an expert in minimally invasive surgery via the tubular system, unilaterally combined with a surgical microscope.

The data were collected in a Microsoft Excel spreadsheet (Microsoft Corporation, Redmond, WA), and key variables were analyzed using IBM SPSS version 29 (IBM Corp., Armonk, NY). Permission was requested from the hospital management to access data from the medical records, and the research protocol was submitted to the Ethics and Research Committee, which approved it under registration number PI-2024-14. Permission to use the Oswestry Disability Index was also obtained through the official platform (https://eprovide.mapi trust.org/instruments/oswestry-disability-index), and its use for this study was authorized [14].

## Results

**Figure 1:**
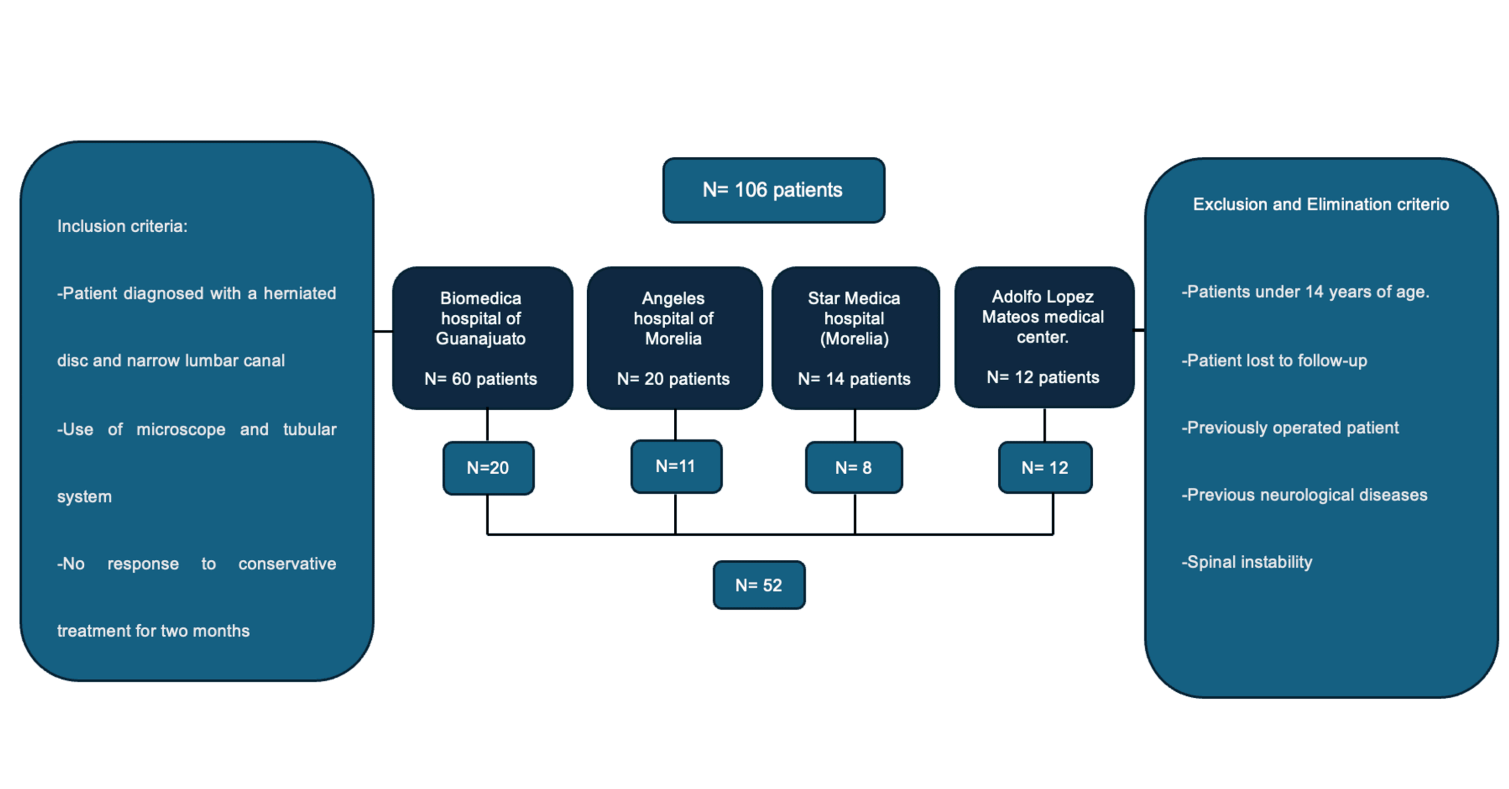
Sample selection criteria. N = Number of patients.

Figure 1 shows the sample selection criteria.

Patient characteristics and surgical outcomes are shown in Table 1. A total of 51 patients were included in this study. The age of the patients in this study ranged from 18 to 81 years, with a mean of 51 years. The sample included 27 male patients (52.9%) and 24 female patients (47.1%), with a greater predominance of the male sex. The vast majority of patients underwent only one spinal procedure.

**Table 1:** Patient characteristics and surgical outcomes. Q: Quartile.

		Median	Q_25_-Q_75_
Age		51	39-66
Number of procedures		1	1-1
Number of levels operated		1	1-2
Surgery time		120	69-138
Surgical bleeding		100	50-150
Length of hospital stay		2	2-3
Preoperative visual analog pain scale		8	7-9
		Number	Percentage
Sex	Male	27	52.9%
	Female	24	47.1%
Diagnosis level	Disc herniation	31	60.8%
	Narrow lumbar duct	20	39.2%
	L4	3	5.9%
	L4-L5	22	43.1%
	L5-S1	14	27.5%
	L3-L4/L4-L5	3	5.9%
	L4-L5/L5-S1	8	15.7%
	L3-L4/L4-L5/L5-S1	1	2.0%
Dural tear	Yes	1	2.0%
	No	50	98.0%
Fistula	No	51	100.0%
	Yes	0	0.0%
Infections	No	50	98.0%
	Yes	1	2.0%
Wound dehiscence	No	51	100.0%
	Yes	0	0.0%
Preoperative Oswestry	Minimal disability	4	7.8%
	Moderate disability	5	9.8%
	Severe disability	17	33.3%
	Crippled	17	33.3%
	Bed-bound	8	15.7%

Among the diagnoses, 31 patients (60.8%) had herniated discs and 20 (39.2%) had narrow lumbar canals. The L4-L5 level (22; 43.1%) was the most commonly affected area, followed by the L5-S1 (14; 27.5%) and L4 (3; 5.9%) levels. Most patients underwent operations in just one level.

The surgical procedures lasted an average of 112 minutes, with a minimum of 45 minutes and a maximum of 258 minutes. Most (50; 98%) patients did not experience dural tears, with just one (2%) presenting with this complication.

The average amount of blood loss was 118 ml, with a minimum of 10 ml and a maximum of 400 ml. No blood transfusions were required for any patient. The mean length of hospital stay was two days, with a minimum of one day and a maximum of seven days. No patient required admission to intensive care. Among the possible complications observed, only one patient (2.0%) experienced a dural tear. There was one case of surgical site infection (2%) and no cases of wound dehiscence.

**Figure 2:**
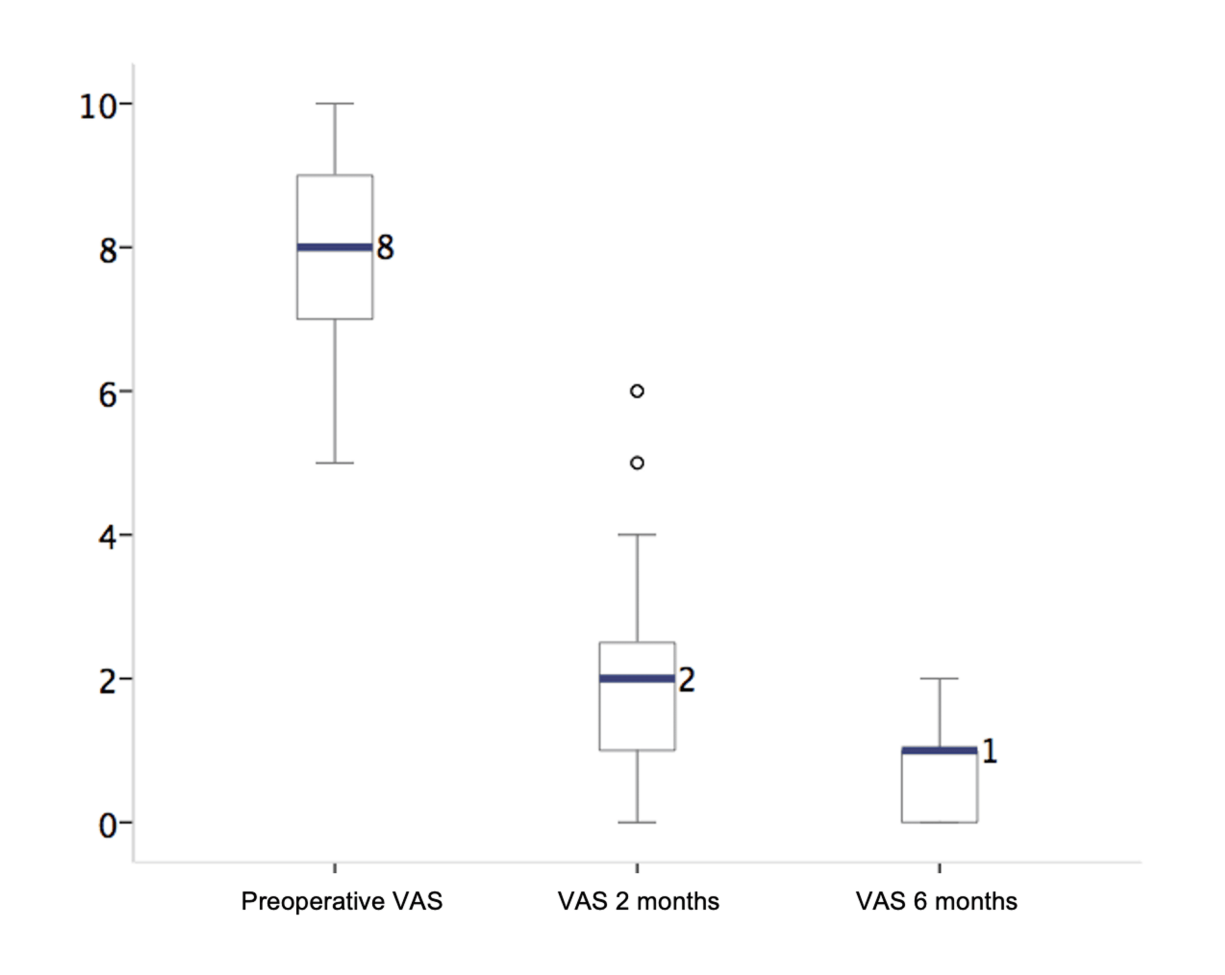
Box-and-whisker plot showing the distribution of quartile data of the visual analog pain scale, highlighting the median in a blue bar and outliers as a circle. VAS: visual analog scale.

**Figure 3:**
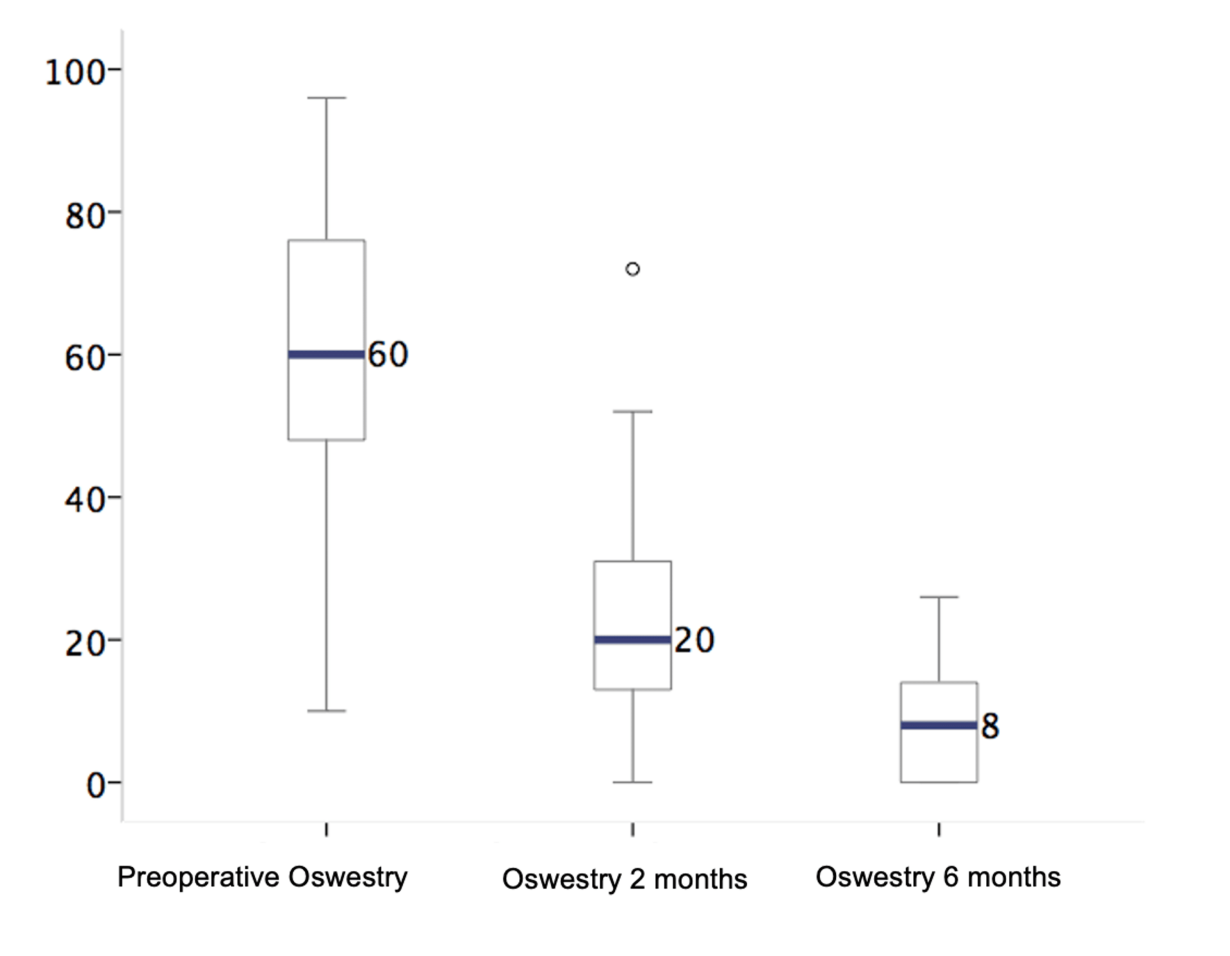
Box-and-whisker plot showing the quartile data distribution of the Oswestry Disability Index, highlighting the median in a blue bar and outliers as a circle.

**Figure 4:**
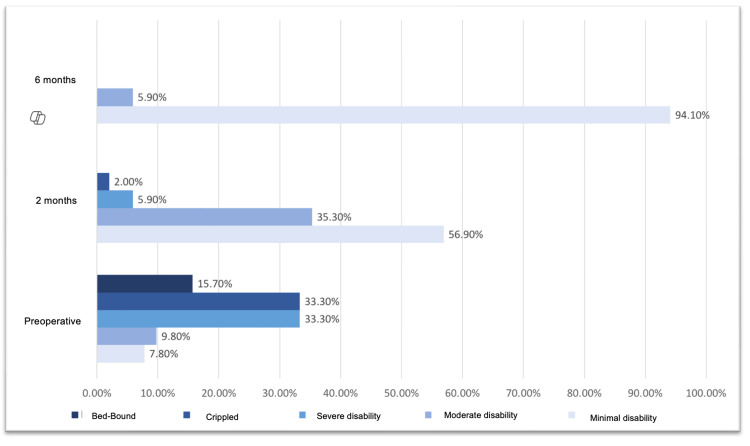
Histogram showing changes in presurgical and postsurgical Oswestry Disability Indices.

The Oswestry Disability Index was used to evaluate functional status, as shown in Table 2, and the VAS was used to evaluate pain. The most common preoperative VAS score among the 51 patients was 8/10. The most frequently observed value at two months after the operation was 2/10. The most frequently observed value at six months after the operation was 1/10. These findings are presented in Figure 2. The Oswestry Disability Index is summarized in Table 3. This table shows a mean percentage of 60% in the preoperative period, with a minimum of 10% and a maximum of 96%. This finding indicates that four (7.8%) patients had minimal disabilities, five (9.8%) had moderate disabilities, and 17 (33.3%) had severe disabilities. There was a presence of total disability in eight (15.7%) cases. Findings from the Oswestry Disability Index at two months post surgery demonstrated an average score of 20%, indicating minimal functional limitations. A total of 8% reported minimal disability at six months after surgery, as shown in Figure 3. Figure 4 shows a histogram summarizing the change in the Oswestry Disability Index scores before and after surgery.

**Table 2:** The 25-75 quartiles and preoperative means of the visual analog pain scale and Oswestry Disability Index at two and six months postoperation. Q: Quartile.

	Preoperative		2 months		6 months		P-value
	Median (Q25-75)		Median (Q25-Q75)		Median (Q25-Q75)		
Visual Analog Scale	8	7-9	2	1-3	1	0-1	<0.001
Oswestry Disability Index	60%	48-76	20%	10-32	8%	0-14	<0.001

**Table 3:** Oswestry Disability Index scores preoperatively and at two and six months. N = Number of patients.

	Preoperative		2 months		6 months	
	n	%	n	%	n	%
Minimal disability	4	7.8%	29	56.9%	48	94.1%
Moderate disability	5	9.8%	18	35.3%	3	5.9%
Severe disability	17	33.3%	3	5.9%	0	0.0%
Crippled	17	33.3%	1	2.0%	0	0.0%
Bed-bound	8	15.7%	0	0.0%	0	0.0%

## Discussion

Lumbar disc disease and lumbar canal stenosis are two of the most common conditions affecting the lumbar spine. Low back pain with associated radicular pain and paresthesia is a cause of significant morbidity, leading to lost workdays and increased healthcare expenditures, with its obvious implications [16,17].

Lumbar canal stenosis is one of the most common degenerative spinal disorders among elderly individuals, with an incidence of 1.7% to 8%. A total of 60% of these individuals have multilevel disorders. Elderly people are being increasingly diagnosed with lumbar canal stenosis, owing to increased life expectancies, the advent of MRI, and demanding lifestyles [18].

Lumbar disc herniation is a pathology that mainly affects adults between 30 and 50 years of age [19]. A narrow lumbar canal is prevalent among individuals over 60 years of age [20]. However, the mean age of individuals affected by this pathology in this study was 51 years. Some studies have shown that lumbar disc herniation is more common in female patients, while others have shown that it is more common in male patients [20-22]. Males were most commonly affected in this study.

A case series conducted in Mexico involving spinal pathology epidemiology showed that the most common type of this disease was a narrow lumbar canal, followed by disc herniation [23]. In that study, the diagnosis of disc herniation was more common than that of a narrow lumbar canal, which differs from our results. Armenta et al. [24] reported that the most affected spinal level is L4-L5, followed by L5-S1. Santiago-Rubio et al. [25] reported that pathology most commonly occurs at two levels, mainly at the L4-L5 level. In this study, the L4-L5 segment was most affected, which aligns with the aforementioned epidemiological case series from Mexico.

Results from a study of the intraoperative characteristics of the tubular approach, which was carried out at Nizam's Institute of Medical Sciences located in India, showed that the average amount of blood loss during surgery was 97.45 ml, the average surgical time was 97.8 minutes, and the average length of post-surgery hospital stay was 1.34 days. In that study, one patient developed severe radiculopathy, while another experienced knee extension weakness and aggravated lumbar and radicular pain after mobilization, along with bladder retention. There were four cases of intraoperative dural tear but no cases of postoperative cerebrospinal fluid leakage [26]. The duration of the surgical procedure was longer in this study, with a mean of 120 minutes. The mean blood loss during surgery was 100 ml, which is similar to that reported by the Nizam's Institute of Medical Sciences. The average length of hospital stay was two days. There was one dural tear without cerebrospinal fluid leakage in the postoperative period and one surgical site infection, which were followed up with outpatient care during which no new complications occurred. The Oswestry scale is one of the most widely used and recommended indicators of disability status worldwide [26], and it has predictive value and is used to evaluate low back pain, sick leave duration, and conservative or surgical treatment results. Kumar et al. [26] conducted a study using the Oswestry Disability Index, finding that the average disability level was 68.19% during the preoperative period and 32% at the six-month follow-up [26]. In this study, the mean preoperative Oswestry scale scores were 60%, 20% at two months, and 8% at six months; these findings contrast with those of Kumar et al. [26], who reported that there was substantial functional improvement in the lumbar spine in patients who received surgical management [26]. Pain intensity, measured using the VAS, is one of the most commonly used outcome measures. Kumar et al. [26] reported mean VAS scores of 7, 2, and 2 immediately after surgery, at three months postoperation, and at six months postoperation, respectively. The mean preoperative VAS score among the 51 patients in our study was 8 points. The mean scores at two and six months postoperatively were 2 and 1, indicating that surgery improved pain status.

This study has limitations. As it is not a randomized, controlled study, there is a risk of selection bias and uncontrolled confounding, which may influence the internal validity of the findings. The lack of a comparative group (patients operated on with open techniques) limits the possibility of establishing direct comparisons that allow assessing the superiority of the tubular approach combined with a microscope. Although the sample is consecutive, its relatively small size reduces the ability to generalize the results to other populations or surgical settings.

## Conclusions

Disc herniation and lumbar spinal canal stenosis represent significant public health concerns, requiring a comprehensive understanding of contemporary diagnostic and therapeutic approaches to ensure timely intervention and optimize clinical outcomes. The use of a surgical microscope in combination with a tubular system has demonstrated effectiveness in the management of these conditions by enhancing anatomical visualization, increasing precision during discectomy and neural decompression, and minimizing trauma to surrounding tissues. These advantages are associated with faster postoperative recovery, shorter hospital stays, and improved postoperative quality of life.

The findings of this study indicate that the tubular system-assisted microscopic technique is safe and effective, with low complication rates and clinically significant improvements in pain and functional status, as assessed using the VAS and the Oswestry Disability Index. Taken together, these results support the integration of this minimally invasive approach as a valuable advancement in modern spine surgery.
